# Reticulocyte Maturation

**DOI:** 10.3390/membranes12030311

**Published:** 2022-03-10

**Authors:** Christian J. Stevens-Hernandez, Lesley J. Bruce

**Affiliations:** 1 Bristol Institute for Transfusion Sciences, National Health Service (NHS) Blood and Transplant, Bristol BS34 7QH, UK; christian.stevens@nhsbt.nhs.uk; 2 School of Biochemistry, University of Bristol, Bristol BS8 ITD, UK

**Keywords:** reticulocyte maturation, red cell variants, red cell membrane proteins, incomplete reticulocyte maturation

## Abstract

Changes to the membrane proteins and rearrangement of the cytoskeleton must occur for a reticulocyte to mature into a red blood cell (RBC). Different mechanisms of reticulocyte maturation have been proposed to reduce the size and volume of the reticulocyte plasma membrane and to eliminate residual organelles. Lysosomal protein degradation, exosome release, autophagy and the extrusion of large autophagic–endocytic hybrid vesicles have been shown to contribute to reticulocyte maturation. These processes may occur simultaneously or perhaps sequentially. Reticulocyte maturation is incompletely understood and requires further investigation. RBCs with membrane defects or cation leak disorders caused by genetic variants offer an insight into reticulocyte maturation as they present characteristics of incomplete maturation. In this review, we compare the structure of the mature RBC membrane with that of the reticulocyte. We discuss the mechanisms of reticulocyte maturation with a focus on incomplete reticulocyte maturation in red cell variants.

## 1. Overview

The mature red blood cell (RBC) has a unique biconcave shape that provides flexibility and withstands shear stress during circulation through narrow blood capillaries. The biconcave shape is maintained by interactions between the RBC membrane and the underlying cytoskeleton [1]. Changes in these horizontal and vertical interactions, or alterations in the surface to volume ratio, state of hydration and/or cell metabolism, destabilize the cell and cause hemolytic anemias [2]. However, despite the wear and tear of the circulation and the high levels of oxidative stress, typically RBCs survive in the circulation for 120 days before they are removed by the spleen. Reticulocytes, on the other hand, are much less stable. Reticulocytes are formed in the bone marrow, resulting from the enucleation of late-stage erythroblasts, and begin as large, multi-lobular, motile cells known as R1 reticulocytes [3]. In order to mature into RBCs, they must lose 20% of their plasma membrane, lose any residual organelles or internal membranes and selectively reduce or remove cytoplasmic and membrane proteins that are not required by RBC [4,5,6]. Exactly how this is achieved is not completely understood, but in this review, we will discuss the mechanisms that are thought to contribute to this process.

## 2. Structure of the Mature RBC Membrane

### 2.1. The Lipid Bilayer

**Figure 1:**
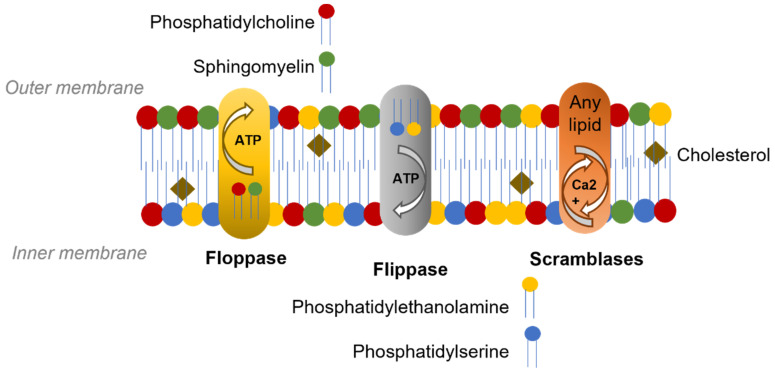
Phospholipids distribution in the RBC membrane. The phospholipids in the RBC membrane have an asymmetric distribution while cholesterol is evenly arranged. Phospholipid location is maintained by the ATP-dependent translocases, flippases transport phosphatidylserine and phosphatidylethanolamine, from the outer to the inner monolayer and floppases transport phosphatidylcholine and sphingomyelin from the inner to the outer monolayer. The Ca^2+^-dependent scramblases can transport phospholipids in both directions.

The gross structure of the red cell membrane is a lipid bilayer with attachments to the underlying cytoskeleton via tethering transmembrane protein complexes. This structure provides the membrane with a dynamic fluidity and elasticity to survive for 120 days during circulation [7]. The lipid bilayer contains an equal distribution of cholesterol and phospholipids. While cholesterol is evenly located, phospholipids have an asymmetric distribution across the two membrane leaflets. The outer membrane leaflet contains mainly the choline phospholipids phosphatidylcholine (PC) and sphingomyelin (SP) while the amino phospholipids, phosphatidylethanolamine (PE) and phosphatidylserine (PS) are confined to the inner leaflet [8,9]. Phospholipids diffuse across the membrane passively; the asymmetry of their distribution is maintained and regulated by specific enzymes, two ATP-dependent translocases and a Ca^2+^-dependent scramblase (Figure 1). Flippases transport PS and PE, from the outer to the inner monolayer, at a higher rate compared to floppases which transport SP and PC from the inner to the outer monolayer [10]. The maintenance of the aminophospholipids (PS and PE) in the inner leaflet may also be aided by their interaction with the cytoskeleton via spectrin [11,12].

Scramblases can rapidly transport phospholipids bi-directionally; however, this enzyme is only active when there is an increase in Ca^2+^ influx which normally occurs in pathophysiological conditions or during mechanical stimulation of the RBC membrane [10,13]. The regulation of phospholipid asymmetry is essential to maintain membrane integrity; uncontrolled phospholipid translocation may result in blebbing of the plasma membrane in the form of PS positive microvesicles. PS exposure is often observed in senescent [14], sickle [15] and *P. falciparum* infected red blood cells [16].

### 2.2. RBC Membrane Proteins

The RBC membrane contains numerous proteins. Many of these are transmembrane proteins, inserted into the lipid bilayer; others are peripheral proteins, attached to the membrane via protein–protein or protein–lipid interactions. Several proteins are inserted into the outer leaflet of the membrane by a glycosyl-phosphatidylinositol (GPI) anchor [17].

**Figure 2:**
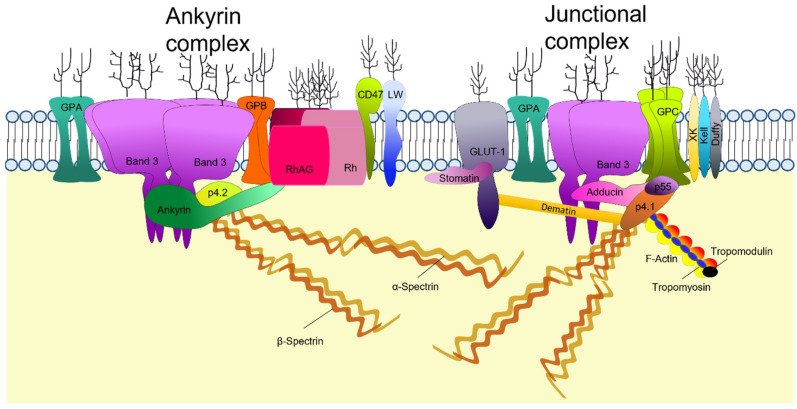
RBC membrane structure. Two main macromolecular complexes anchor the lipid bilayer to the cytoskeleton. The ankyrin complex: Ankyrin binds to band 3 and forms a complex with protein 4.2, Rh, Rh-associated glycoprotein (RhAG), CD47, LW and glycophorins A and B (GPA and GPB). The junctional complex: glycophorin C (GPC), protein 4.1 (p4.1) and p55 bind to actin, dimeric band 3 associates with adducin, and the glucose transporter-1 (GLUT-1) associates with both dematin and stomatin. Actin and numerous actin-binding proteins associate with the α and β-spectrin tetramers.

Proteomic analysis of RBCs membranes has identified over 300 major membrane proteins [18] although the RBC surface proteome shows that 62–75% of the proteins consists of just four proteins, band 3 (anion exchanger 1 (AE1); SLC4A1), glucose transporter 1 (GLUT1), glycophorin A (GPA) and glycophorin C (GPC) [19]. Approximately 25 of the transmembrane proteins carry blood-group antigens and most membrane proteins can be grouped according to their function as transporters, adhesion molecules and signaling receptors for extracellular interactions [20]. Many of the membrane proteins associate in large complexes. The two main protein complexes that maintain the structural integrity of the membrane by forming the major links with the underlying cytoskeleton are the ankyrin complex and the junctional complex (Figure 2). The ankyrin complex attaches the tetrameric form of band 3 to β1-spectrin, an interaction stabilized by protein 4.2 [21]. The ankyrin complex includes other proteins as demonstrated in band 3 null/deficient RBCs where protein 4.2 is absent and spectrin, ankyrin, GPA and proteins from the Rh complex are significantly reduced [22,23]. Band 3 is also present in the lipid bilayer as a dimer, either in a mobile, unattached form or in association with the junctional complex [24]. The junctional complex of proteins attaches GPC to actin, an interaction stabilized by protein 4.1 and p55. The complex is also thought to include dimeric band 3, associated with adducin, GPA, GLUT1 associated with dematin, Kell, XK, the Rh polypeptides, and numerous actin-binding proteins (Figure 2) [25,26]. Adducin and tropomodulin promote the capping of actin filaments and tropomyosin regulates actin filament length [27]. The junctional complex anchors the ends of around six spectrin tetramers forming the hexagonal lattice of the cytoskeleton [28].

### 2.3. Membrane Cytoskeleton

The RBC cytoskeleton comprises a spectrin-based network distributed as a two-dimensional hexagonal lattice, although pentagons (3%) and heptagons (8%) are also observed [28,29]. Spectrin tetramers form the sides of the hexagons and are joined by the junctional complexes. Mature RBCs contain α1-spectrin and β1-spectrin and the functional basic unit of spectrin in the red cell cytoskeleton is the α1/β1-spectrin tetramer. Each single chain of α1-spectrin is comprised of 21 triple helical repeats and a partial repeat (α0); β1-spectrin contains 16 repeats and a single partial repeat (β17). The α1 and β1-spectrin chains intertwine as antiparallel dimers and are able to self-associate at the head end where the partial helix units bind together (α0/β17) to form the spectrin tetramers [30] Repeats 14 and 15 of β1-spectrin bind to ankyrin, and thus to the tetrameric band 3 complex, one main attachment site between the cytoskeleton and the membrane [31]. The tail ends of the spectrin tetramers contain binding sites for actin, protein 4.1 and other associated cytoskeletal proteins of the junctional complex, the other main attachment site between the cytoskeleton and the membrane [32].

In order for the red cell to deform and maintain elasticity, spectrin must be able to dissociate and reassociate. This process is thought to involve the spectrin self-association sites [33]. The attachment between spectrin and ankyrin is very strong and its disruption causes membrane instability and vesiculation [34]. Disruptions to the tail-end spectrin interactions with protein 4.1 and actin using 2,3-DPG likewise cause membrane instability [35]. However, it is suggested that in normal physiological conditions the link between the self-association sites allows the rupture of spectrin tetramers into dimers so that the membrane can adapt to different mechanical forces during circulation [36].

The ability of the membrane to recover its biconcave shape after deformation relies on the lateral cytoskeletal interactions. Actin is a key linker protein; approximately 5–12 spectrin tetramers bind to each actin filament [37] in the junctional complex and the interaction is strengthened by protein 4.1 [38] and other accessory proteins. Aside from the junctional complex the ankyrin associations with band 3 and with spectrin are known to promote the self-association of spectrin tetramers, adding to the equilibrium between intact and dissociated tetramers in the resting membrane. This association is strengthened by the presence of protein 4.2 [21].

## 3. Erythropoiesis

To adequately supply tissues with oxygen, the bone marrow needs to produce 2 million erythrocytes per second; however, RBC production can be tailored to the body’s demand. In certain pathological conditions the regulatory network is overloaded, resulting in the excessive production of RBCs or anemia [39].

**Figure 3:**
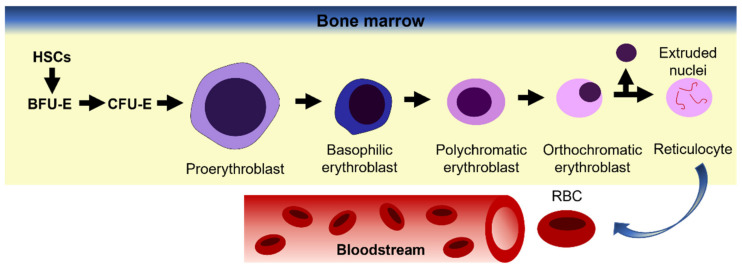
Erythropoiesis model. Hemopoietic stem cells (HSC) resident in the bone marrow proliferate and differentiate into burst-forming units (BFU-E) which further differentiate into highly proliferative colony forming units (CFU-E). Further differentiation gives rise to the earliest erythroblast progenitors, proerythroblasts. Maturation of erythroid progenitors is marked by changes in cell size, hemoglobinization, increased chromatin condensation and finally enucleation. The resultant cell is a reticulocyte which is released into the circulation to complete the final stage of maturation into an erythrocyte. Adapted from Shah, Huang and Cheng [46].

At the stage of definitive erythropoiesis in mammals, terminal differentiation occurs in the fetal liver, thymus and spleen; later it progresses to the bone marrow. During gestation, hematopoietic stem cells (HSCs) migrate to the bone marrow where they remain quiescent and provide the source of erythropoiesis for the post-natal life [40]. The bone marrow acts as a microenvironment for the interaction of HSCs with growth factors and cytokines that promote the proliferation and formation of burst-forming-unit-erythroid cells (BFU-e) (Figure 3). Erythropoietin, a kidney-derived erythropoiesis regulator, then promotes the differentiation of BFU-e into rapidly dividing colony-forming-unit erythroid cells (CFU-e) [41]. The next stage of terminal differentiation begins with the formation of erythroblastic islands where a central macrophage interacts with up to 30 pro-erythroblasts through the binding of adhesion molecules and receptors [42]. Over the next 2–3 days, erythroid cells follow their fate from pro-erythroblasts down through the basophilic, polychromatic and orthochromatic stages [43]. During these last stages the cells decrease in size, nuclear chromatin is condensed and hemoglobinization occurs [44]. The final terminal differentiation events constitute the expulsion of the nucleus, which is phagocytosed by the resident macrophages and release of the nascent reticulocyte into circulation where it completes maturation into an erythrocyte over the next 1–2 days [45] (Figure 3).

### 3.1. Membrane Protein Changes and Assembly during Terminal Erythroblast Differentiation

The expression of membrane proteins is dynamic and different populations of erythroblasts at different developmental stages can be resolved by determining the distinct protein expression patterns during erythropoiesis. Early murine and rabbit models suggest that the large structural membrane proteins are synthesized at the late erythropoiesis stage and act as anchors to smaller membrane and cytoskeletal proteins [47,48,49]. One early study of erythropoiesis, studying terminal differentiation in erythroblasts from Friend virus (FV)-infected mouse spleens, showed that erythroid cells synthesize band 3 and protein 4.1 at the early pro-erythroblasts and basophilic stages of erythropoiesis [50]. These results were also observed in other erythropoiesis study models such as rabbit and rat-derived nucleated precursors and in vivo mouse studies [47,48,49,51].

Studies of human erythropoiesis in cell culture systems have shown that synthesis of the red cell membrane proteins occurs in an unsynchronized manner [52,53]. As CD34 declines, the expression of proteins that are found in the mature RBC membrane increases. Some of the first to appear are Kell, LW, Rh associated glycoprotein (RhAG) and GPA, next comes band 3 and RhCE, followed by Lutheran, RhD and Duffy [52]. These studies used flow cytometry and only detected some of the antigen-carrying membrane proteins. It has since been shown that expression of other membrane proteins increases similarly, including GLUT1. GLUT1, in association with stomatin, is key to the uptake of ascorbic acid as an oxidant buffer for the high iron content in young progenitors and also participates in glucose transport [54].

Newly synthesized spectrins are highly abundant in the cytoplasm even earlier, at early erythroblast stages, but much of this protein is turned over and only a small fraction associates with the membrane. As maturation progresses spectrin synthesis declines, however, the proportion of membrane-bound spectrin is increased [51]. The decline in spectrin synthesis and increased membrane-associated spectrin is observed alongside high band 3 and protein 4.1 production. At this point, hemoglobin synthesis is also upregulated [55]. It is suggested that band 3 and protein 4.1 are required for the stabilization of the spectrin proteins and the assembly of the spectrin-actin-protein 4.1 junctional complex with actin mirroring the production pattern of spectrin [47]. Newly synthesized ankyrin in the cytosol undergoes rapid degradation until later maturation where the mRNA of different ankyrin transcripts are expressed, resulting in products with variable binding affinities that may be adaptive to different functional requirement during development [5,48]. Ankyrin-1 is required for the formation of tetrameric band 3 and forms the core of the ankyrin macrocomplex (Figure 2). The ankyrin protein complex assembly during erythropoiesis is band 3 dependent. Protein associations of band 3 and protein 4.2 begin in the Golgi and ER during the basophilic stage prior to incorporation into the plasma membrane [56]; however, whether stabilization of ankyrin occurs before or after its incorporation into the membrane is still subject to debate [57,58]. Contrary to the expression pattern of the major membrane proteins such as band 3, GPA and GLUT1 that increase during erythroid differentiation, adhesion molecules such as CD44, α4, α5 and β1 integrins decrease [59]. In vitro derived human erythroblasts can be sorted into an α4+ population of nucleated erythroid cells and an α4- population of reticulocytes [60]. The loss of adhesive proteins is predisposed by the protein rearrangement that erythroblasts undergo prior to enucleation.

### 3.2. Protein Rearrangement during Erythroblast Enucleation

During the terminal differentiation stage, orthochromatic erythroblasts undergo enucleation where they expel their nucleus and become reticulocytes. It is unclear what initiates the process of enucleation; however, it is agreed that it is a multi-stage operation. It is characterized by increased transcriptional activity for specific erythroid genes, including α and β-globins [61], chromatin condensation and a reduction of the nuclei to a tenth of its original volume [62].

At the end of the last cell cycle, polarization of the nucleus to one side adjacent to the plasma membrane is observed. The downregulation of vimentin occurs towards the final stages of erythroblast differentiation. It is suggested that the removal of vimentin allows the nucleus to be freely mobile to move towards the plasma membrane and begin the process of enucleation [63]. In other non-mammalian species, where nucleated erythrocytes are in circulation, vimentin anchors the nucleus [64].

Various comparisons have been made between the process of cytokinesis and enucleation [65]. Once the nucleus is polarized, phase microscopy shows the plasma membrane forming a constriction around the nucleus forming a cleavage furrow, similar to that in mitotic cytokinesis, separating the nuclei and the nascent reticulocyte. Mitochondria are observed to accumulate around the area of constriction, probably providing the energy required to expel the nucleus. Actin has also been observed to accumulate around the cleavage furrow in the form of a contractile ring and effective enucleation is prevented with cytochalasin D, a cytokinesis inhibitor that binds to actin [66,67]. Furthermore, the pan-inhibitor for Rac-GTPases also suppressed enucleation [66]. It was suggested that Rac-GTPases not only promote the polymerization of actin to form a contractile ring but also associate with lipid rafts that are merged within the furrow, both events necessary for the activation of signaling pathways that lead to enucleation [68].

Early electron microscopy images from canine blood have shown the formation of vesicles adjacent to the mitochondria, between the plasma membrane and the nuclear membrane [69]. It is suggested that these vesicles are mobilized across the cytoplasm with the aid of myosin V and VI, two actin-dependent motor proteins. Once these vesicles are at the cleavage furrow, they fuse together to form coalescence vesicles that provide the replacement plasma membrane for the area that will be removed with the nucleus. This allows the separation of the nascent reticulocyte and the nucleus, which is surrounded by plasma membrane derived from the original cell [70].

A differential distribution of cytoskeletal and membrane proteins occurs during enucleation. Spectrin staining on enucleating erythroblasts from mouse bone marrow is absent from the membrane surrounding the extruding nucleus (pyrenocyte) but present in the reticulocyte; in contrast, actin is present in both regions [71]. Similar results were observed in human erythroblasts enucleated in vitro. Cytoskeletal and cytoskeletal-associated proteins such as ankyrin, adducin, protein 4.1 [72] and protein 4.2 are seen in reticulocytes; however, actin and p55 are distributed across both entities. Certain membrane proteins, which are believed to have been synthesized in excess and therefore discarded during enucleation, are band 3 and glycophorin C; major protein losses also occur for stomatin, CD44 and Glut-1 [73,74]. Proteomic analysis of reticulocytes compared to the extruded pyrenocytes shows that pyrenocyte fractions contain high levels of endoplasmic reticulocyte proteins such as calnexin and calreticulin as well as nuclear proteins such as histones. As for the reticulocyte fraction, in addition to cytoskeletal and membrane proteins, peptides from cytosolic enzymes and endocytic proteins were also identified [73].

Survivin has been shown to interact with endocytic vesicle traffic mediating proteins EPS15 and clathrin. The loss of survivin by knockdown and deletion studies demonstrated enucleation inhibition and lack of visible cytoplasmic vacuoles and the presence of autophagosomes that contained un-degraded organelles. It is suggested that survivin may also play a role with endo-lysosomes and autophagolysosomes to orchestrate selective protein trafficking and degradation during enucleation [75,76].

A protein that is key during hemoglobinization in erythroid terminal differentiation is the transferrin receptor (TfR). In early erythroblasts, TfR is highly expressed in the membrane and may be seen in patches all around the cytoplasm. Later during differentiation, orthochromatic erythroblasts stain for TfR in the region adjacent to the extruding nucleus where intracellular vesicles accumulate prior to enucleation [70,77]. The inhibition of endocytosis by MitMAB (a dynamin inhibitor) on mouse spleen derived erythroblasts prevented the accumulation of TfR on the plasma membrane at the enucleation site, hampering enucleation and hemoglobin synthesis [78,79]. During hemoglobinization, TfR follows the endosomal recycling pathway; however, once hemoglobin synthesis is complete TfR expression is downregulated to prevent iron-mediated toxicity [80]. Aggregation of TfR and other molecules such as acetylcholinesterase promotes the switch from a recycling fate to the removal of obsolete proteins via the exocytic pathway [81].

## 4. Mechanisms of Reticulocyte Maturation

Reticulocyte maturation begins after enucleation in the bone marrow. In rats, reticulocytes reside in the bone marrow from 6.5–17 hrs depending on the blood demand [34]. These reticulocytes are termed as R1 and are characterized for their multi-lobular shape and their motility. The final stages of maturation occur during circulation where macrophages residing in the spleen may facilitate the process [82]. These reticulocytes in circulation are termed as R2, are non-motile and have a “deep-dish” shape” [3]. As part of their maturation, reticulocytes need to remove or degrade residual organelles and RNA [83,84]. In addition, the reticulocyte must reduce its surface area and volume. On average, labeled baboon reticulocytes showed a reduction of 20% of their surface area and 15% of their volume after the first 24 h in circulation; at this point they showed a similar size distribution to that of mature RBC [4].

### 4.1. Protein Removal through Exosome Release

**Figure 4:**
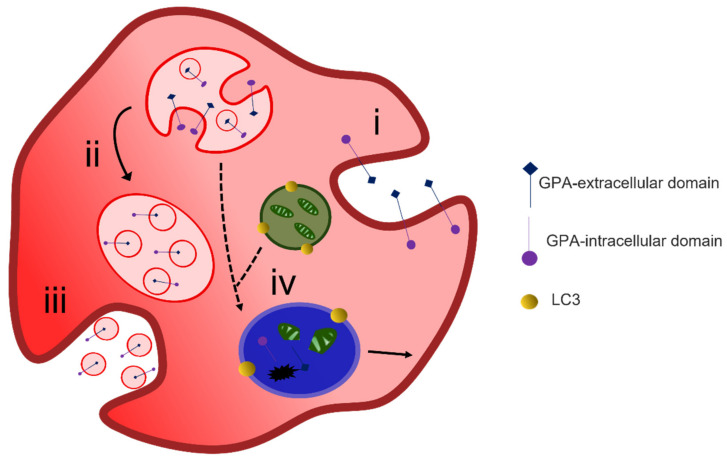
Exocytosis-mediated reticulocyte maturation. During reticulocyte maturation unwanted membrane proteins are endocytosed (i) and sorted into multivesicular bodies (ii) that are released as exosomes via the fusion of MVB membrane with the cell plasma membrane (iii). LC3-positive autophagosomes containing degraded organelles may also fuse with MVB and lysosomes (iv) and form autophagolysosomal vacuole where cargo degradation occurs or alternatively exits the cell via membrane budding.

Multiple mechanisms have been shown to contribute to the process of reticulocyte maturation. Johnstone, Bianchini and Teng [85] and Pan and Johnstone [86], described the externalization of TfR in the form of vesicles during the maturation of sheep reticulocytes. They showed that this was achieved by a process of endocytosis of TfR from the surface of the plasma membrane into the cytoplasm. These newly formed endosomes fused together to form larger vesicle structures. Budding at the internal surface of the large vesicles resulted in the formation of multivesicular bodies (MVB) ranging from 0.5–1 μm in diameter, with intra-luminal vesicles (ILV) of ~50 nm in diameter [87] The MVB would fuse with the plasma membrane releasing the internal vesicles termed exosomes (Figure 4).

Characterization of the exosomes released has shown that the removal of obsolete proteins is a selective process. Compartmentalization of exosome-derived proteins from cord blood reticulocytes showed that the main protein fractions were from the cytosol 15.9%, the plasma membrane 14.9% and the nucleus 13.5%. In equal parts (~5%) proteins were derived from the cytoskeleton, lysosomal, endosomal and Golgi compartments. Lower protein numbers were from the ER and the mitochondria [88].

The sorting of membrane and cytosolic proteins into exosomes and not into the lysosomal pathway has been attributed to parallel mechanisms. The first one involves the binding of the cytosolic proteins Alix and heat shock cognate 70 to ubiquitinated proteins forming a link with the endosomal sorting for complex transport machinery (ESCRT, I and II). ESCRT III then promotes the formation of ILVs by assisting in pit formation and budding off the membrane [89,90]. Budding of exosomes from the intraluminal membrane of MVB has also been attributed to an ESCRT-independent system where lipid-raft micro-domains are formed in areas with ceramide enrichment [91].

A second mechanism is also initiated by aggregation of receptors which has been shown to prevent rejoining the recycling pathway and the clusters may act as signals for exosome processing [81]. Aggregation has also been promoted by the addition of exogenous lectins. Galectin-5 is removed from the surface during maturation of rat reticulocytes; this lectin is shown to enter the endosomal compartment from the cytosol and associate with the ILV surface. Lamp2, an endo-lysosomal protein which is also lost during reticulocyte maturation, has a binding motif for Galectin 5. Galectin-5 has also been shown to play a role in the sorting of glycoproteins into exosomes [92]. Lipidomic studies on exosomes have suggested that all these pathways occur sequentially with the involvement of ceramides within lipid rafts featuring at the later stages of maturation [89].

### 4.2. Alternative Methods of Reticulocyte Maturation

**Figure 5:**
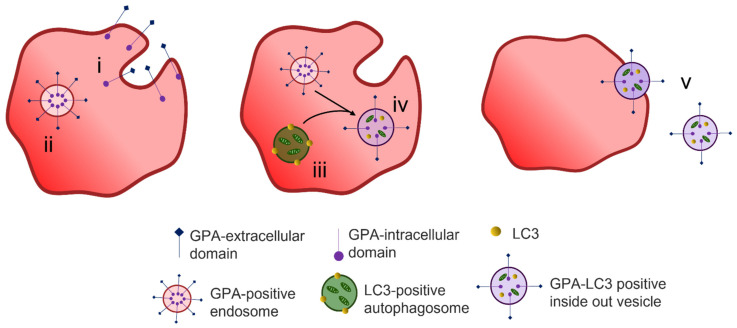
Reticulocyte maturation by expulsion of inside out hybrid vesicles. Endocytosis of superfluous membrane proteins occurs at the plasma membrane (i) during reticulocyte maturation forming GPA-positive endosomes (ii). LC3-positive autophagosomes are formed from isolated membranes that engulf cytoplasmic content including organelles (iii). The GPA-positive endosomes fuse with LC3-positive autophagosomes forming hybrid LC3-GPA positive inside out vesicles (iv) that are extruded during passage through the spleen (v). Figure modified from Mankelow et al. [94].

The process of exosome release to remove unwanted proteins does not fully explain the reduction in plasma membrane surface area during maturation of a reticulocyte to an erythrocyte. A two-stage alternative reticulocyte maturation mechanism has been proposed. In the bone marrow, the formation of an R2 reticulocyte from an R1 reticulocyte follows the removal of redundant proteins through the endosome–exosome pathway. For the final maturation, the R2 must remove excess plasma membrane. The plasma membrane is taken into the cytosol by endocytosis, and the GPA-A containing endosome fuses with an autophagosome which is positive for the autophagosomal marker LC3; together they form an inside-out vesicle that is extruded through the plasma membrane at an area of weakened cytoskeleton [93,94] (Figure 5). These inside-out vesicles are phosphatidylserine positive and stain with markers for organelles such as calreticulin for the ER, giantin for the Golgi and MitoTracker for the mitochondria [95]. Their extrusion through the plasma membrane is thought to be driven by non-muscle myosin II [96].

Another model for membrane loss focusing on the role of membrane rafts for the latter stage of reticulocyte maturation has been described [97]. Membrane rafts are known to be rich in cholesterol and sphingolipids. During early reticulocyte maturation these lipid rafts are gradually lost; however, differential expression of some membrane raft markers is observed in the mature erythrocyte membrane. This is suggested to be due to the presence of different type of lipid rafts. Areas of the membrane associated with flotillin-rich lipid rafts bulge and are removed by macrophages during circulation, resulting in removal of plasma membrane. Areas containing stomatin-rich lipid rafts are maintained within the membrane and allow anchoring to the cytoskeleton, which contributes to the formation of the final biconcave shape of a mature erythrocyte [98].

### 4.3. Organelle Clearance

Autophagy is believed to be the system by which organelle clearance occurs. Autophagocytosis begins with the formation of a C-shaped membrane known as a phagophore that originates in the endoplasmic reticulum (ER), the mitochondria or the Golgi [99]. During reticulocyte maturation, phagophore assembly is regulated by the autophagy-related proteins (Atg), in particular, Atg5 and Atg7. Although Atg5/Atg7-independent autophagy process has been shown to be involved for mitochondrial and ribosomal clearance, Ulk1 (Atg1)-deficient mice appear to retain mitochondria compared to wild type and Atg5-deficient mice [100]. This Ulk1-dependent pathway appears to rely on regulatory Ras-associated binding proteins (Rab) that are involved in endosome fusion, transport, degradation and recycling [101].

The role of the phagophore is to surround organelles due for elimination and enclose them in a double-membrane vesicle known as an autophagosome [102]. The method of the autophagosome clearance remains controversial. In the canonical pathway the autophagosome fuses with lysosomes for cargo degradation by the action of hydrolytic enzymes. However, in erythroid cells, there is evidence of removal of the lysosomal compartment by disappearance of LAMP2 by exocytosis [92]. As previously mentioned, a method of organelle elimination, combined with membrane reduction, was proposed to occur by the formation of large inside out hybrid vesicles that are extruded from the membrane to be removed by splenic macrophages [95]. Alternatively, the interaction of autophagosomes with MVB has been proposed as a model for organelle clearance. In K562 cells, endocytic vesicles label with the autophagosome marker LC3 [103,104] and electron microscopy of rat reticulocytes shows that they contain TfR positive internal vesicles that have membrane continuity with autophagic vacuoles [105].

## 5. Mature Red Blood Cell versus Immature Red Blood Cell Membrane

Recent proteomic studies of human erythropoiesis have identified protein differences between the mature RBC and the reticulocyte. Absolute quantification of the reticulocyte proteome identified 1658 proteins with a similar or higher expression compared to RBCs; 654 proteins were reticulocyte specific and are probably removed during reticulocyte maturation [106]. Proteins that are highly active in reticulocytes but reduced or absent from mature RBCs mainly correspond to those involved in protein translation, protein regulation and nucleic acid binding, while the proteomic content of mature RBCs is highly associated with lipid and oxygen binding, the main function of a circulating RBC [106,107].

It is interesting that proteins that provide anchoring points between the membrane and the cytoskeleton for mechanical stability (e.g., band 3, Rh, RHAG, GPC and XK) appear to increase after maturation [106,108]. The increased protein levels of the major membrane complex proteins may be due to re-localization of these proteins from the cytosol into the membrane [108] or the agglomeration of proteins in the membrane, as a result of cholesterol loss during reticulocyte maturation [89]. The enrichment of these proteins may also occur because their attachment with the cytoskeleton prevents their removal during the maturation process. Vesicles derived from maturing reticulocytes do not contain these major anchoring membrane proteins; instead they are enriched in proteins with no cytoskeletal linkages such as CD59 [109]. Levels of other proteins with looser cytoskeletal links such as GLUT4, CD47, Kell and Na-K ATPase are reduced during maturation [107]. However, it is argued that membrane protein retention may not be due to the cytoskeletal linkages but the association of membrane proteins with different lipid rafts [110].

These differences in protein profiles between the immature and the mature membrane may explain the increased stability of RBCs compared to reticulocytes. Ektacytometer assays, where deformability is measured, show that low shear stress allows the formation of ellipse-shaped mature cells, but a much higher shear stress is required to promote deformation and prevent axial rotation of the immature reticulocytes [111]. The increased stability of the mature membrane allows the reversal to its original biconcave shape and prevents fragmentation, observed during the aspiration experiments in immature reticulocytes [112,113].

In addition, reticulocytes have a highly active protein kinase [114] and more phosphorylated peptides are derived from reticulocytes when passed through a micro-filtration system compared to RBCs [115]. Phosphorylation regulates cytoskeletal and membrane interactions which contribute to membrane stability. An increased serine and threonine phosphorylation status has also been observed in cytoskeletal proteins from stored RBCs where membrane stability is decreased [116].

The shear modulus of reticulocytes, which represents the elastic stiffness of the membrane, gradually decreases during reticulocyte maturation; R1 reticulocytes have a shear modulus of 11.4 pN/μm, R2 reticulocytes 6.1 pN/μm [112] and a mature RBC is 7.4 ± 0.9 pN/μm when measured by diffraction phase microscopy [117]. The reduced deformability from the reticulocytes may be due to the tensile stress experienced by the cytoskeleton due to the excess membrane. Similarly, RBCs from patients with red cell membrane disorders appear to have decreased mechanical stability and therefore shortened survival, resulting in hemolytic anemia [118]. Recent studies have shown that some variant RBCs not only have decreased mechanical stability but also fail to complete reticulocyte maturation normally [119,120].

## 6. Red Cell Membrane Variants

There are numerous variants that affect the structure and interactions of proteins of the RBC membrane and thus the deformability and stability of the cell. The inter-dimer spectrin interactions and the lateral (horizontal) linkages between spectrin and the proteins of the junctional complex provide mechanical stability to the membrane. Weakening of these networks may result in decreased membrane integrity leading to hereditary elliptocytosis (HE) and hereditary pyropoikilocytosis (HPP) [121]. To our knowledge, the degree of reticulocyte maturation in HE and HPP RBCs has not been reported. It is thought that, rather than a defect in reticulocyte maturation, it is the continuous traversing the inter-endothelial slits in the spleen that causes irreversible elongation of the HE RBC, which leads to the eventual formation of an elliptical shape or, in the most severe cases, results in cell fragmentation and lysis as seen in HPP RBCs [122].

When the vertical interactions between the membrane and the cytoskeleton are weakened, plasma membrane may be lost by vesiculation reducing the RBC surface area and leading to hereditary spherocytosis (HS). Hereditary spherocytosis (HS) is the most common non-immune hemolytic anemia across ethnic groups. This inherited disorder is caused by either autosomal dominant or autosomal recessive mutations in structural RBC membrane proteins, leading to protein deficiency or loss of function. The most common cause in Caucasian populations (40–65%) are mutations in ankyrin, while in Japan a missense mutation of protein 4.2 is found widely (45–50%). Null or deficient phenotypes for band 3 (20–35%) and the spectrins (α < 5%, β15–30%) account for the remainder of HS cases [123]. In HS the RBC undergo membrane loss through vesiculation and the discocyte shape eventually becomes a spherocyte with reduced deformability [124]. There is some evidence that reticulocyte maturation proceeds normally in HS RBCs, at least in the band 3 deficient cells [120]. The heterozygous HS RBC membranes appear to clear residual mitochondrial, lysosomal, endoplasmic reticulum (ER) and obsolete proteins to the same extent as standard RBCs [120]. The loss of membrane, causing spherocytosis, seems to occur post-maturation and during the circulation of the HS RBC.

The RBC membrane stability and morphology are also dependent on hydration. The volume of healthy RBCs is regulated by the transport of Na+ and K+ cations across the plasma membrane maintaining an osmotic gradient. Autosomal dominant disorders, known as the hereditary stomatocytosis (HSt) group, affect the cell cation permeability, which in turn alters cell morphology and stability [125]. These conditions include dehydrated hereditary stomatocytosis (DHSt) and overhydrated hereditary stomatocytosis (OHSt), familial pseudohyperkalaemia (FP), cryohydrocytosis (CHC), stomatin-deficient cryohydrocytosis (sdCHC) and South-east Asian Ovalocytosis (SAO) [118].

In contrast to the HE, HPP and HS RBCs, some of the HSt RBCs do seem to have defective reticulocyte maturation. It has been shown that RBCs of the OHSt, CHC, sdCHC and SAO phenotypes all show some degree of incomplete reticulocyte maturation [120]. Interestingly, the degree of the maturation defect is reflected by the mean cell volume (MCV) of the variant RBC. OHSt RBCs are larger and less mature than CHC, sdCHC, and SAO RBC which are larger and less mature than standard RBCs. OHSt is caused by heterozygous variants in the Rh-associated glycoprotein (RhAG) [126]. OHSt RBCs show the greatest change in membrane permeability, being 40 times more permeable to monovalent cations than standard RBCs, and have a MCV of about 140 fL [127,128]. This is very similar to the MCV of reticulocytes and cultured red blood cells (cRBCs, cultured reticulocytes), i.e., reticulocytes cultured from CD34+ cells in vitro [93]. Immunoblotting analysis of OHSt RBC membranes compared to cRBC or standard RBC membranes showed that the protein profile of OHSt membranes was more similar to that of cRBCs than standard RBCs, suggesting that OHSt RBCs had achieved minimal reticulocyte maturation [120].

sdCHC is caused by heterozygous variants in SLC2A1 and these RBCs have an increased membrane permeability about 10 times greater than normal and a MCV of about 120 fL [129,130]. Immunoblotting analysis of sdCHC RBC membranes as compared to cRBC or standard RBC membranes showed that the protein profile of these membranes was similar to that of standard RBCs, but the sdCHC membranes contained more calreticulin, an ER marker, and more TfR, proteins that are usually decreased during reticulocyte maturation, suggesting that sdCHC RBCs mature to a greater extent than OHSt RBCs but still have incomplete maturation relative to standard RBCs [120].

CHC and SAO are caused by heterozygous variants in SLC4A1 and these cells have an increased membrane permeability of about 2–4 times greater than normal. The MCVs of CHC and SAO RBCs are often within the normal range, but the RBCs contain varying numbers of stomatocytes, and these variant cells tend to be larger, about 100–120 fL [119,131]. Immunoblotting analysis of CHC and SAO RBC membranes, as compared to cRBC or standard RBC membranes, showed a similar protein profile to the sdCHC RBC membranes, except for the TfR in the SAO cells, and CD147 in CHC cells, suggesting that CHC and SAO RBCs also present incomplete maturation when compared to standard RBCs, but mature to a greater extent than OHSt cells [120]. To our knowledge, the degree of reticulocyte maturation in DHSt RBC has yet to be analyzed. It is not anticipated that FP RBCs will have a reticulocyte maturation defect as FP is a benign, asymptomatic condition [132]. Together the study of reticulocyte maturation in HSt variant RBCs provided an insight into the reticulocyte maturation process and a better understanding of the effect of HSt variants on RBC maturation.

## 7. Conclusions

In this review we have discussed the structure of the mature RBC membrane and how it differs from the membrane of the reticulocyte. The processes of erythropoiesis and enucleation have been touched upon and the various mechanisms of reticulocyte maturation discussed. These mechanisms, lysosomal protein degradation, exosome release, autophagy and the extrusion of large autophagic-endocytic hybrid vesicles, probably occur simultaneously to a certain extent. However, there is increasing evidence that these mechanisms occur at different time points and in different environments. Lysosomal protein degradation is a standard process for the removal of unwanted or damaged proteins in nucleated cells and probably occurs even before enucleation of the erythroblast. Exosome release may well occur predominantly in the bone marrow islands, where macrophages can immediately engulf the exosomes. It would surely cause a problem if circulating reticulocytes released millions of exosomes into the blood serum. The formation of the large hybrid vesicles that are extruded through the RBC membrane is probably the final stage of reticulocyte maturation. These vesicles can be seen protruding from RBCs in the circulation of people with defective spleens. The hybrid vesicles seem to require splenic conditioning in order to be extruded and this process probably requires an interaction with a macrophage. The hybrid vesicles are not simply released into the blood serum. Interestingly, the study of the variant RBCs seems to bear out this timeline with the mitochondria, lysosomes and some of the CD147 and TfR being lost as the MCV reduces from 140 fL to 110 fL. Then the ER membranes and remaining CD147 and TfR are lost as the MCV reduces from 110 fL to about 90 fL. Of course, this is just speculation and exactly how reticulocyte maturation is achieved is not completely understood; further study is needed to determine the precise mechanisms and timeline.

## Data Availability

Not applicable.
